# The prognostic value of zonal origin in clinically localized prostate cancer: a systematic review and meta-analysis

**DOI:** 10.3389/fonc.2023.1248222

**Published:** 2023-12-08

**Authors:** Shijie Jin, Liyi Wu, Zhen Liang, Weigang Yan

**Affiliations:** Department of Urology, Peking Union Medical College Hospital, Beijing, China

**Keywords:** prostatic neoplasms, transition zone, peripheral zone, prognosis, biochemical recurrence

## Abstract

**Introduction:**

Correlation between zonal origin of clinically localized prostate cancer (PC) and biochemical recurrence (BCR) after treatment is still controversial.

**Methods:**

We performed a meta-analysis of published articles to investigate the prognostic value of zonal origin in clinically localized PC. Literature was searched from Medline, Embase, Scopus, and Web of Science, from inception to Nov 1st, 2022. The risk of BCR was compared between PC originating from transition zone with peripheral zone. Relative risk (RR) was pooled in a random-effects model. Subgroup analysis and meta-regression were conducted to assess the source of heterogeneity.

**Results:**

16 cohorts and 19,365 patients were included. PC originating from transition zone was associated with a lower risk of BCR (RR, 0.79, 95%CI; 0.69-0.92, I^2^, 76.8%). The association was consistent in studies with median follow-up time ≥60 months (RR, 0.65; 95%CI, 0.48 to 0.88, I2 56.8%), studies with NOS score ≥8 (RR, 0.70; 95%CI, 0.62 to 0.80, I^2^ 32.4%), and studies using multivariate regression model (RR, 0.57; 95%CI, 0.48 to 0.69, I^2^ 23%).

**Discussion:**

This meta-analysis supported that transition zone origin was an independent prognostic factor of a better biochemical result in clinically localized prostate cancer after treatment.

**Systematic review registration:**

10.37766/inplasy2023.11.0100, identifier INPLASY2023110100.

## 1 Introduction

Prostate cancer (PC) is the second most common cancer in men and the sixth most common cause of cancer death worldwide in 2020, causing >350,000 death in men (1). In most men, prostate cancer is diagnosed while the disease is confined within the prostate (2), which is termed localized prostate cancer. Initial treatment of clinically localized PC includes radical prostatectomy (RP), radiotherapy (RT), hormonal therapy, and deferred treatments such as active surveillance and watchful waiting (3–5). However, after RP or RT with curative intent, up to 27–53% of these patients experience biochemical recurrence (BCR) (6). BCR is considered as an early event indicating disease progression and is related to a higher risk of metastasis and disease-specific mortality. To estimate the risks of BCR, D’Amico classification system was developed and verified. Now the derivatives of D’Amico system are widely used in clinical practice (3–5). However, the best stratification and optimal treatment remain controversial (5).

The human prostate was histologically divided into transition zone (TZ), peripheral zone (PZ), central zone (CZ), and anterior fibromuscular stroma (AFMS) by McNeal (7). Approximately 25%, 70%, and 5% of prostate cancer originate respectively from TZ, PZ, and CZ (8). Heterogeneity has been found between prostate cancers with different zonal origins. Compared with PZ tumors, most TZ tumors are usually diagnosed with larger volume and higher prostatic specific antigen (PSA) levels, but with earlier T stage and lower Gleason scores, indicating that TZ tumors might have better biological behavior. Some studies suggested that zonal origin in TZ was associated with a lower risk of BCR (9, 10). Conversely, other studies found no significant differences in 5-year biochemical relapse-free survival between TZ tumors and PZ tumors (11). Information about CZ tumors is limited due to scarcity (12). Therefore, the prognostic role of zonal origin in prostate cancer is still controversial.

Considering that most of the previous studies are retrospective single-institutional, we aim to conduct a meta-analysis of all eligible published studies to quantify the prognostic value of zonal origin in prostate cancer.

## 2 Methods

### 2.1 Search strategy

We conducted this meta-analysis according to the Meta-analysis of Observational Studies in Epidemiology (MOOSE) guidelines and Preferred Reporting Items for Systematic Reviews and Meta-analysis (PRISMA) guidelines (13, 14). The Medline, Embase, Scopus, Web of Science, and Cochrane databases were searched from inception to November 1st, 2022 for human studies investigating the association between zonal origin and BCR in prostate cancer. The main search terms included: (zone or zonal) and (prostate or prostatic) and (cancer carcinoma) and (recurrence or failure or relapse). The reference lists of retrieved articles were also checked for relevant articles.

### 2.2 Eligibility criteria

Inclusion criteria for selecting the studies were as follows: (i) The diagnosis of prostate cancer was pathologically confirmed; (ii) Zonal origin was defined as the zone which contains most part of the index tumor with the highest Gleason score; (iii) Correlation of zonal origin with BCR was reported. Exclusion criteria were the following: (i) Abstracts, letters, case reports, reviews, or nonclinical studies; (ii) Studies were not written in English; (iii) Studies with insufficient data for estimating relative risk (RR), odds ratio (OR), hazard ratio (HR), or 95% confidence interval (CI); (iv) Studies with duplicate data. Initial screening of the title and abstract, full-text assessment, and subsequent data extraction were independently performed by two authors (SJJ and LYW). Disagreements were discussed and resolved by consensus with a third reviewer (ZL).

### 2.3 Data extraction and quality evaluation

The following items were extracted from each included study: authors, year of publication, country, the proportion of different ethnic groups, study design, number of cases, treatment, follow-up time, the definition of zonal origin, the definition of BCR, and confounding factors which were balanced or adjusted. RR, HR, or OR were directly extracted from literature, or indirectly estimated from Kaplan-Meier curves according to the methods illustrated by Parmer et al, together with the 95% CI (15). If results of both univariate and multivariate Cox regression analysis were reported, we chose the multivariate model for a more accurate estimate. We used RR to represent various effect estimates. A RR <1 indicated a better prognosis for prostate cancer originating in transition zone. To evaluate the methodological quality and grade the evidence of included studies, the Newcastle–Ottawa Scale (NOS) (range 1–9 scores) was used (16). NOS scores of ≥8 were defined as high-quality studies.

### 2.4 Statistical analysis

We pooled RRs and 95% CIs using random-effects models and fixed-effects models according to the heterogeneity evaluated by Cochran’s Q test and Higgins I-squared statistic (17). An I^2^ > 50% was considered as significant heterogeneity and a random-effects model (DerSimonian–Laird method) was used. Otherwise, the fixed-effects model (Mantel–Haenszel method) was adopted (18). A subgroup analysis was performed based on variables including major ethnic group, sample size, median follow-up time, regression model type (univariate or multivariate), RR source (direct extraction or indirect estimate), NOS total score, the definition of BCR, the definition of TZ origin (on MRI or pathological sections), pre-treatment PSA level, the ratio of Gleason grade group ≥2, and the ratio of T stage ≥T3. Sensitivity analysis was conducted by omitting one study at a time, generating the pooled estimates, and comparing them with the original estimates. Funnel plots, Begg’s test, and Egger’s test were performed to assess publication bias (19, 20). All analysis was performed using STATA/SE 12.0 (STATA, College Station, TX). Statistical significance was defined as two-tailed alpha <0.05.

## 3 Results

### 3.1 Study selection and characteristics

**Figure 1:**
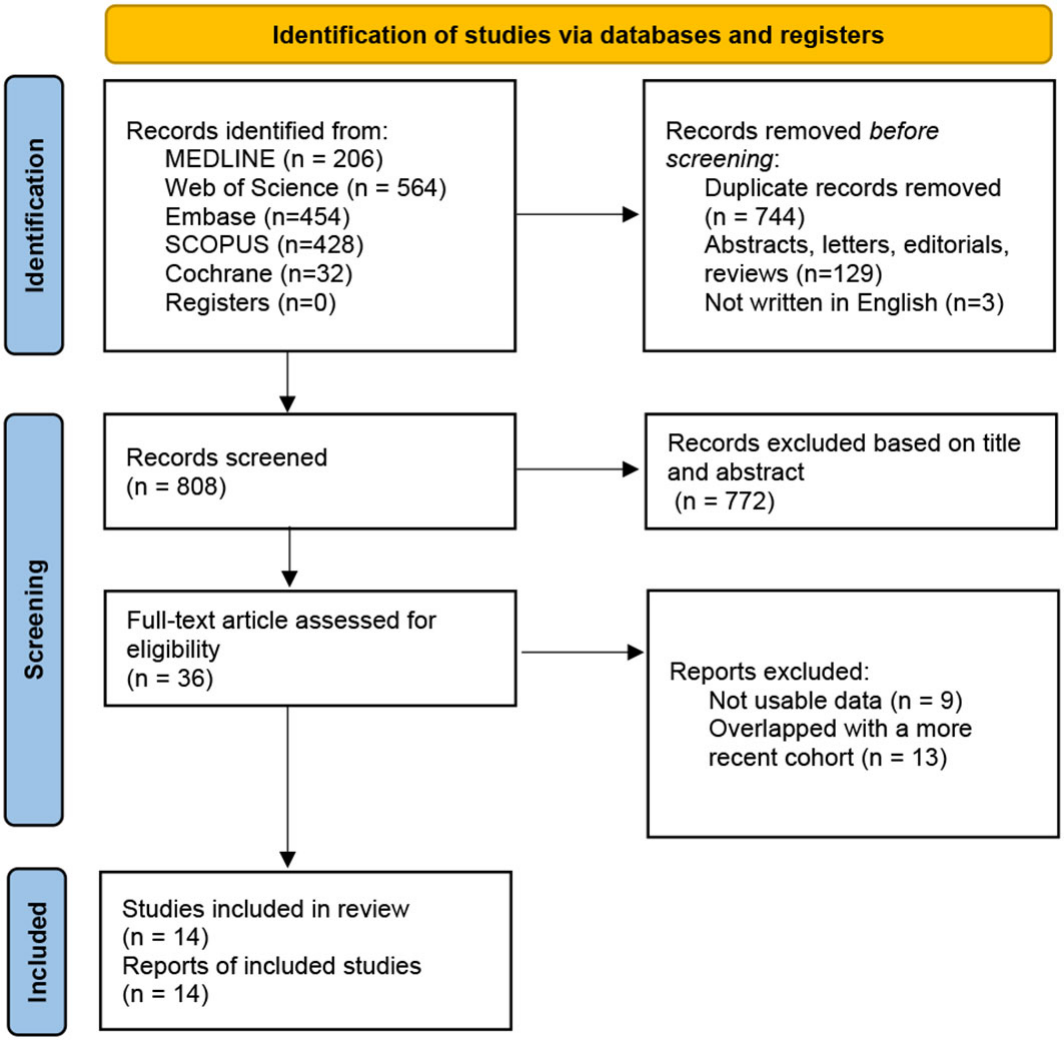
The PRISMA flow chart of the study selection process (13). PRISMA, Preferred Reporting Items for Systematic Reviews and Meta-Analyses.

As shown in **Figure 1**, the literature search initially identified 1684 papers. 36 studies were included in the full-text assessment. Finally, 14 studies, published from 2000 to 2022, were enrolled in the final mete-analysis (9, 21–33). Among these, 13 were cohort studies and 1 was case-control. Studies were conducted in France (n=1), Germany (n=1), Brazil (n=1), USA (n=5), Korea (n=2), Japan (n=3), and Australis (n=1). Since Kim and Teloken’s studies both contained two distinct cohorts, there were 16 cohorts in the meta-analysis. Their detailed characteristics are listed in **Table 1**; **Table S1**.

**Table 1:** Characteristics of included studies.

Studies	Year	Country	Cohort size	Treatment	Follow-up (months)	Definition of TZ tumors	Ratio of TZ tumors	Definition of BCR (PSA messured as ng/ml)	RRs ^a^	Counfounders balanced or adjusted ^b^	NOS score
Asuncion (21)	2022	France	230	RT	104.4	MRI	11.1	nadir + 2	RR(U,I)	N	7
Billis (22)	2017	Brazil	345	RP	45.9	PS	31.9	0.2	HR(U,D)	12568	8
Chun (23)	2007	Germany	1262	RP	45.1	PS	9.1	0.1	RR(U,I)	7	7
Falzarano (24)	2020	USA	485	RP	14.1	PS	27.2	0.2	OR(U,I)	1238	9
Iremashvili (25)	2012	USA	1441	RP	42	PS	11	0.2	HR(M,D)	1235678	9
Kim1 (26)	2020	Korea	1521	RP	N	PS	29.7	N	HR(U,D)	1	4
Kim2 (26)	2020	Korea	2302	RP	N	PS	29.7	N	HR(U,D)	1	4
Lee (27)	2015	USA	1354	RP	84	PS	17	0.1	HR(M,D)	12345678	9
Magheli (28)	2007	USA	265	RP	81.6	PS	19	0.2	RR(U,I)	134578	8
Mygatt (29)	2014	USA	1528	RP	90	PS	10.1	0.2	RR(U,I)	12	8
Sakai (30)	2006	Japan	134	RP	37	PS	20.1	0.2	RR(U,I)	123678	9
Sato (31)	2021	Japan	270	RP	93.8	PS	34.4	0.2	HR(M,D)	123478	9
Shin (32^)^	2020	Korea	232	RP	18	MRI	27.6	0.2	HR(M,D)	345	7
Takamatsu (33)	2019	Japan	638	RP	59	PS	46	0.2	HR(M,D)	123478	9
Teloken1 (9)	2017	Australia	2677	RP	31.8	PS	10.2	0.2	HR(M,D)	134568	8
Teloken2 (9)	2017	Australia	4374	RP	39.8	PS	25.1	0.2	HR(U,D)	67	7

TZ, transition zone; BCR, biochemical recurrence; PSA, prostatic specific antigen; RT, radiation therapy; RP, radical prostatectomy; MRI, magnetic resonance imaging; PS, pathological sections; N, not available.

a Type of relative risk and its source: RR, relative risk; OR, odds ratio; HR, hazard ratio; U, univariate analysis; M, multivariate analysis; D, directly extracted from the text; I, indirectly estimated from the text or Kaplan-Meier curve.

b Confounders balanced between TZ/PZ group in baseline data and counfounders adjusted in multivariate regression analysis: 1, age; 2, T stage; 3, Gleason score/Gleason grade/ISUP group; 4, PSA level before treatment; 5, extraprostatic expansion; 6, seminal vesicle invasion; 7, lymph node involvement; 8, positive surgical margin.

Among the 16 cohorts, sample sizes ranged from 134 to 4374, with a median of 950. The median/mean age ranged from 58.7 to 68.5. The median follow-up time ranged from 18 months to 104.4 months. The ratio of TZ tumors ranged from 9.1% to 46%, and PZ tumors from 54% to 90.1%. CZ tumors were not separately reported in 14 cohorts. In the other 2 cohorts, CZ tumors were excluded from the prognostic analysis. As a result, we can only compare the prognosis of TZ tumors and PZ tumors. Regarding the reported data on prognostic indicators, the median pre-treatment PSA level ranged from 5.7 to 32.2 ng/ml. The ratio of prostate cancer with Gleason grade ≥2 ranged from 34.8% to 100%. And the ratio of prostate cancer with pathological T stage ≥ T3 ranged from 25.5% to 49.3%. Patients undertook RP in 15 cohorts and RT in 1 cohort. The use of neoadjuvant or adjuvant therapy were summarized in **Table S2**. Most RP cohorts excluded patients who received neoadjuvant or adjuvant therapy.

### 3.2 Risk of bias and quality assessment

The methodological quality profile of included studies according to NOS is shown in **Table S3**. The mean NOS score was 7.625. Only one study showed a high risk of bias because it was conference literature and lacked detailed methodological data (26). The most common problems identified were the lack of adjustment for potential confounders. 6 studies used a multivariate regression model, in which confounders were adjusted, such as age, T stage, Gleason grade group, and positive surgical margin. The definition of BCR and zonal origin were not exactly the same, but most studies used PSA level ≥ 0.2 ng/ml as the cut-off for BCR. And most studies define TZ origin when TZ contains more than 70% of the index tumor.

### 3.3 Overall analysis

**Figure 2:**
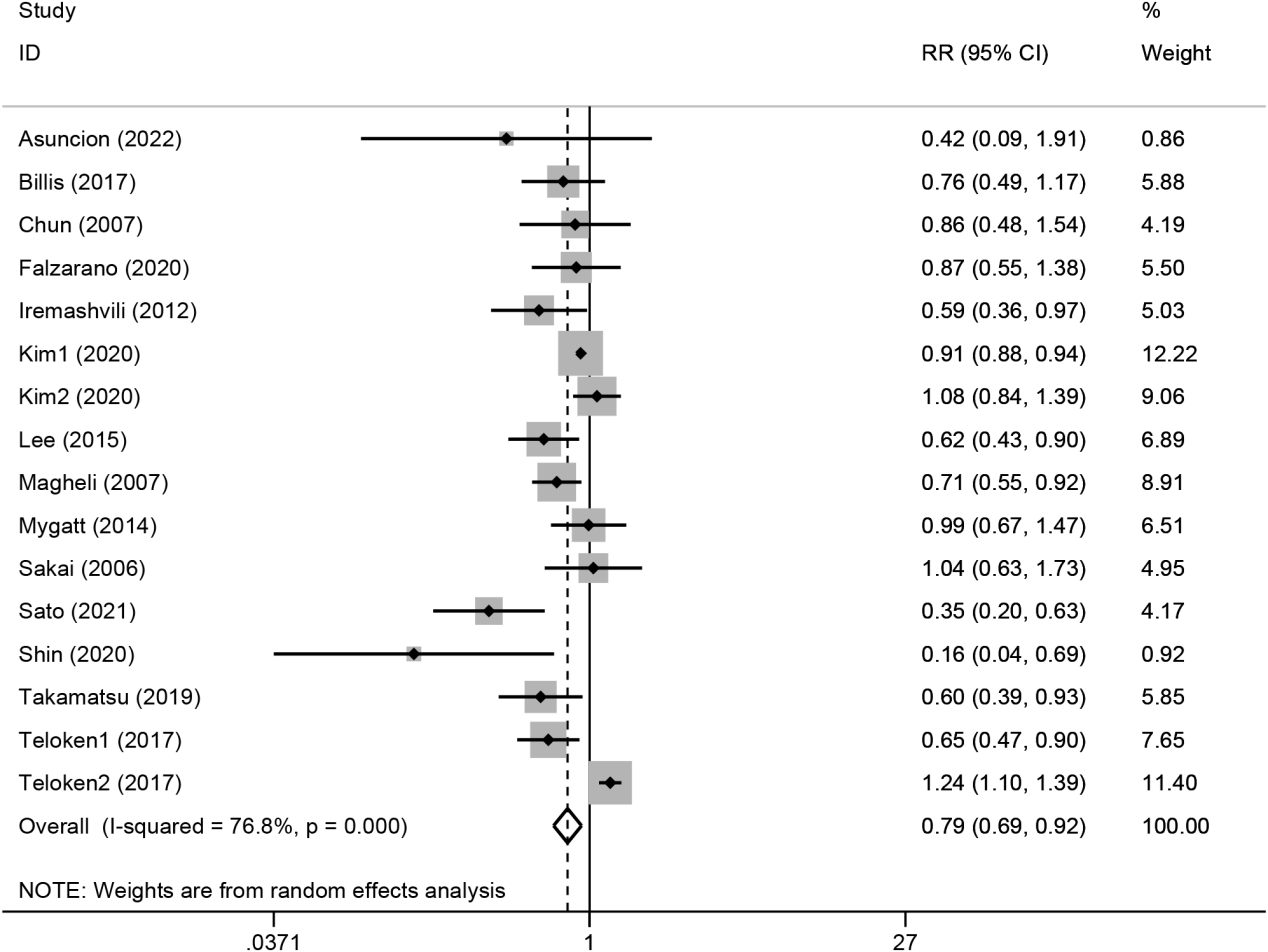
Forest plot of the association between transition zonal origin and biochemical recurrence. Diamonds represent study-specific relative risks or summary relative risks with 95% CIs. Horizontal lines represent 95% CIs. A RR<1 represents better prognosis of TZ tumors.

Because the heterogeneity test showed a high level of heterogeneity (I^2 =^ 76.8%, p<0.01) between the studies, a random-effects model was used for the analysis (see **Figure 2**). A pooled RR of 0.79 (95%CI, 0.69-0.92; p<0.01) showed that clinically localized PC originating from the transition zone were associated with a better outcome in terms of biochemical-free survival.

### 3.4 Subgroup analysis and meta-regression

Given the high heterogeneity showed through the I^2^ statistic, a subgroup analysis and a meta-regression were performed based on the following variables: major ethnic group (Caucasian or eastern Asian), sample size (<1000 or≥1000), median follow-up time (<60m or ≥60m), regression model type (univariate or multivariate), RR source (directly extraction or indirectly estimate), NOS total score(<8, ≥8), BCR definition (0.1 ng/ml or 0.2 ng/ml), definition of TZ origin (on MRI or pathological sections), PSA level (<10 ng/ml or ≥10 ng/ml), ratio of Gleason grade group ≥2 (<80% or ≥80%), and ratio of T stage ≥T3 (<40% or ≥40%). In the subgroup analysis shown in **Table 2**, regardless of the grouping variables used, I^2^ of the subgroups could not drop below 50% at the same time. In the meta-regression shown in **Table 3**, regression model type (P=0.002) and NOS score (P=0.025) were found to be possible sources of heterogeneity, but the residual I^2^ was still high than 50% (64.45% for regression model type and 69.79% for NOS score), indicating that they could only explain part of the heterogeneity.

**Table 2:** Subgroup analysis of the pooled association of transition zonal origin with biochemical recurrence.

Analysis	N	Reference	Random-effects model		Fixed-effects model		Heterogeneity	
			RR	95%CI	RR	95%CI	I^2^	Ph
Subgroup1:Caucasian	10	(9, 21–25, 27–29)	0.79	0.62-1.00			77.8	<0.001
Eastern Asian	6	(26, 30–33)	0.75	0.56-1.00			76.6	0.001
Subgroup2: Sample size <1000	8	(21, 22, 24, 28, 30–33)	0.66	0.51-0.85	0.69	0.59-0.81	48.4	0.059
Sample size ≥1000	8	(9, 23, 25–27, 29)	0.89	0.75-1.05			82	<0.001
Subgroup3: Median follow-up time <60m	9	(9, 22–25, 30, 32)	0.77	0.58-1.03			78.4	<0.001
Median follow-up time ≥60m	5	(21, 27–29, 31)	0.65	0.48-0.88			56.8	0.055
Subgroup4: Univariate	10	(9, 21–24, 26, 28–30)	0.94	0.82-1.09			72.6	<0.001
Multivariate	6	(9, 25, 27, 31–33)	0.56	0.45-0.70	0.57	0.48-0.69	23	0.261
Subgroup5: RR directly extracted	10	(9, 22, 25–27, 31–33)	0.76	0.63-0.92			84.9	<0.001
RR indirectly estimated	6	(21, 23, 24, 28–30)	0.82	0.69-0.98	0.82	0.69-0.98	0	0.584
Subgroup6: NOS score < 8	6	(9, 21, 23, 26, 32)	0.98	0.78-1.23			84.9	<0.001
NOS score ≥ 8	10	(9, 22, 24, 25, 27–31, 33)	0.70	0.60-0.82	0.70	062-0.80	32.4	0.149
Subgroup7: BCR with cut-off of 0.1ng/ml	2	(23, 27)	0.68	0.50-0.93	0.68	0.50-0.93	0	0.351
BCR with cut-off of 0.2ng/ml	11	(9, 22, 24, 25, 28–33)	0.73	0.56-0.95			82.1	<0.001
Subgroup8: TZ origin defined on MRI	2	(21, 32)	0.25	0.09-0.73	0.25	0.09-0.73	0	0.369
TZ origin defined on pathological section	14	(9, 22–31, 33)	0.81	0.71-0.94			77.7	<0.001
Subgroup9: median/mean PSA < 10ng/ml	9	(9, 21, 22, 24, 25, 27, 31, 33)	0.68	0.49-0.94			84.4	<0.001
median/mean PSA > 10ng/ml	6	(23, 26, 28–30)	0.91	0.83-0.99	0.91	0.88-0.94	14.8	0.319
Subgroup10: Ratio of Grg≥2: <80%	6	(21, 23, 25, 26, 29, 30)	0.93	0.76-1.14			15.0	0.318
Ratio of Grg≥2: ≥80%	9	(9, 24, 26–28, 31–33)	0.74	0.60-0.90	0.95	0.80-1.13	86.2	<0.001
Subgroup11: Ratio of T stage≥T3: <40%	3	(22, 29, 33)	0.78	0.58-1.04	0.78	0.61-1.00	28.8	0.246
Ratio of T stage≥T3: ≥40%	3	(24, 30, 31)	0.69	0.37-1.29			76.6	0.014

N, number of cohorts; RR, relative risk; CI, confidence interval; BCR, biochemical recurrence; TZ, transition zone; PSA, prostatic specific antigen; Grg, Gleason grade group.

**Table 3:** Meta-regression analysis for exploring potential sources of heterogeneity.

Variables	Univariate analysis		
	Coefficienct	P	95%CI
Major ethnic group: Eastern Asian v.s. Gaucasion	-0.059	0.768	-0.477to0.360
Sample size: Larger v.s. Smaller	0.279	0.128	-0.091to0.650
Follow-up time: ≥60m v.s. <60m	-0.239	0.250	-0.672to0.192
Multivariate RR v.s. Univariate RR	-0.528	0.002	-0.820to-0.236
RR was directly extracted v.s. RR was indirectly estimated	-0.134	0.509	-0.559to0.290
Total NOS score: ≥8 v.s. <8	-0.358	0.025	-0.663to-0.052
Definition of BCR: 0.2ng/ml v.s. others	-0.120	0.554	-0.547to0.306
Definition of TZ origin: MRI v.s. pathological sections	0.094	0.685	-0.413to0.601
Median/mean PSA level: ≥10ng/ml v.s. <10ng/ml	0.256	0.148	-0.104to0.616
Ratio of Grg≥2: ≥80% v.s. <80%	-0.202	0.344	-0.645to0.242
Ratio of T stage≥T3: ≥40% v.s. <40%	-0.095	0.801	-1.070to0.881

CI, confidence interval; BCR, biochemical recurrence; TZ, transition zone; PSA, prostatic specific antigen; Grg, Gleason grade group.

Results of specific subgroup analysis were consistent with the overall analysis, supporting the prognostic value of TZ origin. The pooled RR was 0.79 (95%CI, 0.69 to 0.92) in studies with long follow-up time (median follow-up time ≥60m), in a random-effects model (I^2^, 76.8%). The pooled RR was 0.70 (95%CI, 0.62 to 0.80) in high-quality studies (NOS≥8), in a fixed-effects model (I^2^, 32.4%). When we restricted the meta-analysis to studies using a multivariate regression model only, the pooled RR was 0.57 (95%CI, 0.48 to 0.69) in a fixed-effects model (I^2^, 23%). Another valuable finding of subgroup analysis was that in the higher Gleason grade subgroup (ratio of Grg≥2 was higher than 80%), the pooled RR was 0.74 (95%CI, 0.60 to 0.90), in contrast with 0.93(95%CI, 0.76 to 1.14) in the lower Gleason grade subgroup (ratio of Grg≥2 was lower than 80%), both in random-effects models (see **Figure S1**).

### 3.5 Sensitivity and publication bias analysis

The sensitivity analysis (see **Figure S2**; **Table S4**) confirmed the stability of the association between zonal origin and BCR because the pooled RR remained stable if a certain cohort was omitted. For example, if we left out the cohort ‘Kim1’ with the highest weight (12.22%), the pooled RR turned out to be 0.75 (95% CI, 0.61 to 0.93), which was even more significant. The funnel plot (see **Figure S3**), Begg’s test, and Egger’s test did not indicate the existence of obvious bias. Pr>|z| was 0.392 for Begg’s test and P>|t| was 0.128 for Egger’s test.

## 4 Discussion

### 4.1 Principal findings

In this meta-analysis, pooling all available data to estimate the prognostic value of zonal origin in clinically localized prostate cancer, we found that patients with prostate cancer originating from the transition zone have a lower risk of BCR compared with patients with prostate cancer originating from the peripheral zone (RR, 0.79; 95%CI, 0.69-0.92). The result was robust in sensitivity analysis and no publication bias was observed. This association should be considered cautiously as there was high heterogeneity in the overall analysis (I^2^, 76.8%). Nevertheless, it was supported by subgroup analysis in high-quality literature with NOS ≥8 (RR, 0.70; 95%CI, 0.62-0.80; I^2^, 32.4%). To our acknowledgment, this was the first meta-analysis about the prognostic value of zonal origin of clinically localized prostate cancer. Our results suggest that the association of zonal origin with BCR merits consideration.

### 4.2 Possible mechanisms for principal findings

Previous studies had indicated the difference between prostate cancer originating from different zones. In patients receiving prostatectomy (9, 12, 21–33), the ratio of TZ tumors ranged from 10% to 30%, compared with a nearly 70% ratio of PZ tumors. Most studies showed that TZ tumors had higher PSA levels, larger volumes, and a higher positive rate of the surgical margin. On the contrary, the pathological stage and Gleason grade of TZ tumors were similar or even better than PZ tumors. The difference in PSA level and tumor volume might be explained through diagnosis delay of TZ tumors because most of the included cohorts used transrectal systematic biopsy, in which the detection of TZ tumors was more difficult (34). Therefore, TZ tumors tend to grow larger and give rise to higher PSA levels when diagnosed. The difference in pathological T stage could be explained by spatial location and prostate histology. TZ tumors are originally far away from the seminal vesicle and the prostate capsule. What’s more, there is an interstitial band around the transition zone which may limit the spread of TZ tumor (35). As a result, it is more difficult for TZ tumors to invade outside the prostate capsule. However, the difference on Gleason grade has not been not well explained.

Regarding the prognosis after treatment with curative intent, conclusions were not consistent. One explanation for this phenomenon is that most of the previous studies did not control confounding factors such as tumor stage, Gleason grade group, and surgical margin. Augustin (10) and O’Neil (36) found that when confounding factors were controlled, there was no difference in 5-year BFS between TZ and PZ tumors. However, other multivariate regression analyses (25, 27, 31–33) support TZ origin as an independent prognostic factor. In our meta-analysis, when T stage and Gleason grade were balanced, the pooled RR was 0.57 (95%CI 0.48-0.69), indicating that TZ tumors might be a different clinical entity with better prognosis, regardless of T stage and Gleason grade. It is worth noticing that the studies of Augustin (10) and O’Neil (36) were not included in our final analysis because of new literature from the same center (9, 23).

Molecular biology might help to reveal the fundamental differences between TZ and PZ tumors. Adler (37) used genome-wide oligonucleotide microarray on micro-dissected normal prostate tissues from TZ and PZ. They found 351 significant differentially expressed genes. The most significantly highly expressed genes in PZ were mostly targets of ETGs, a transcription factor family which is associated with prostate cancer. Al Kadhi’s metabonomics study (38) indicated that the pathway associated with lipid biosynthesis, with was considered a contributor to prostate cancer, was significantly enhanced in PZ. Guo (39) and Falzarano (40) found that the TMPSSR2-ERG fusion event, which was common in prostate cancer, was more frequent in PZ tumors. The expression of Ki-67, MMP-2, MMP9, p53, and Bcl-2 was also less observed in TZ cancer by Lee (27). Based on those findings, more mechanistic studies are needed to connect the molecular difference with prognosis.

### 4.3 Secondary findings

One interesting finding in our meta-analysis was the different prognostic role of TZ origin between the high Gleason grade group and the low Gleason grade group. In Kim’s (26) and Teloken’s (9) study, prostate cancer was divided into the low-grade group (Grg 1/2) and the high-grade group (Grg 3/4/5). They both found that the prognostic value of TZ origin was only significant in the high-grade group. Our subgroup analysis had similar results, as shown in **Figure S1**. In cohorts with more than 80% of patients having Grg ≥ 2, the pooled RR was 0.74 (95%CI, 0.60-0.90), while in cohorts with less than 80% patients having Grg ≥ 2, the pooled RR was 0.93 (95%CI, 0.76-1.14). These findings suggest the prognostic value of TZ origin might be more worthwhile in prostate cancer with a high Gleason grade. Those intermediate or high-risk patients with TZ tumors might should receive less adjuvant treatment to avoid adverse effects. However, this conclusion needs more support because there are only two studies focusing on the high-grade group, and in most of the included cohorts, patients who received adjuvant treatment were excluded. In our literature search, no literature compared the oncological outcome of prostate cancer originating from transition or peripheral zone who received adjuvant treatment.

Another point worth discussing is that two of our included studies (21, 32) used lesion location on MRI to define TZ tumor. The definition of zonal origin through MRI or pathological sections was not a source of heterogeneity and the prognostic value of TZ origin was consistent in the subgroup using MRI or pathological sections. This supports the use of MRI because it is more practical and non-invasive. A few studies have investigated the ability of MRI to diagnose the location of tumors. In Shin’s study (32), tumor location was verified through pathology, and the concordant rate between MRI and pathology was 86.2% (200/232). In Goldman’s study of 64 men, the overall correlation was 89.1% (41). In Wilbulpolprasert’s cohort of 415 patients, using whole-mount histopathology as a reference, the sensitivity was 79.1%(246/311) for PZ tumors and 73.1%(76/104) for TZ tumors (42). Overall, lesion location on MRI might be acceptably sensitive to predict tumor location on pathological examination but more research is needed.

### 4.4 Limitations

Our study had several limitations. First was the heterogeneity. Though we used subgroup analysis and meta-regression, only the regression model type were identified as possible sources of heterogeneity and they could only explain a limited part of the heterogeneity. Secondly, RRs were indirectly estimated from 6 cohorts, which might introduce errors. However, subgroup analysis showed that the prognostic value was still significant and not changed much when we considered the source of RRs. Thirdly, only one study of radiation therapy and no study of active surveillance were included, because there is a lack of relevant literature. As a result, our result was unsuitable for patients who received radiation therapy or active surveillance. We expect relevant research to fill this gap in the future. Finally, there was a language bias, since our search included only studies written in English. In the future, we plan to focus on the prognostic value of zonal origin specifically in high-grade group prostate cancer, and include high-quality studies to decrease the heterogeneity.

## 5 Conclusion

In conclusion, our study supports that tumor location is an independent prognostic indicator of BCR after radical prostatectomy and is promising to be included in the postoperative risk stratification system. Prostate tumors originating from the transition zone might be a different clinical entity with a better prognosis. The biological mechanisms behind such correlation remained partially unclear, and thus better designed epidemiological and mechanistic studies were necessary to clarify the underlying mechanism. More radiation therapy cohorts and active surveillance cohorts are also desperately needed to verify the prognostic value of zonal origin in such patients.

## Data availability statement

The original contributions presented in the study are included in the article/**Supplementary Material**. Further inquiries can be directed to the corresponding author.

## Author contributions

SJ, LW, and WY were responsible for the conception and design of the review. SJ, LW, and ZL contributed to the data acquisition and interpretation. SJ and ZL were responsible for data analysis. SJ, LW, and ZL contributed to the drafting of the manuscript. WY contributed to data interpretation and revised the paper critically in terms of argument. All authors contributed to the article and approved the submitted version.
