# Supplementary material for: The prognostic value of zonal origin in clinically localized prostate cancer: a systematic review and meta-analysis
Source: Front Oncol. 2023 Dec 8;13:1248222. doi: 10.3389/fonc.2023.1248222 (PMC10739310; doi:10.3389/fonc.2023.1248222)
Supplement: Supplementary file 1 [file DataSheet_1.docx]

Supplementary Material

The prognostic value of zonal origin in clinically localized prostate cancer: a systematic review and meta-analysis

Shijie Jin, Liyi Wu, Zhen Liang, Weigang Yan^*^

*** Correspondence:** Weigang Yan: yanweigang@pumch.cn

## TableS1. Supplementary characteristics of included studies

| Study | Proportion of ethnic group | Medain age | Median pre-treatment PSA level (ng/ml) | Ratio of ISUP≥2 | Ratio of T stage ≥T3 |
| --- | --- | --- | --- | --- | --- |
| Asuncion | N | 68 | 7.5 | 34.8 | N |
| Billis | Whites: 80.1%  Black-Brazilians：19.9% | 63.13 | 8.98 | N | 25.5 |
| Chun | Mainly Whites | N | 11.2 | 59.5 | N |
| Falzarano | White: 86.4%  African American: 10.3%  Others: 3.3% | 59 | 6.04 | 86.6 | 40.4 |
| Iremashvili | N | 60.9 | 5.7 | 58 | N |
| Kim1 | Mainly Eastern Asian | 66 | 12.7 | 100 | N |
| Kim2 | Mainly Eastern Asian | 66 | 12.7 | N | N |
| Lee | Mainly Eastern Asian | 62.5 | 8.41 | 80.7 | N |
| Magheli | White: 86%  Black: 13%  Others: 1% | 58.7 | 32.2 | 80 | N |
| Mygatt | White: 70.5%  Black: 26.7%  Others: 2.8% | 60 | 10.5 | 46.2 | 36.9 |
| Sakai | Mainly Eastern Asian | 68.5 | 13.5 | N | 49.3 |
| Sato | Mainly Eastern Asian | 65 | 8.15 | 85.6 | 45.6 |
| Shin | Mainly Eastern Asian | 64.5 | N | 92.2 | N |
| Takamatsu | Mainly Eastern Asian | 65 | 6.7 | 89 | 32 |
| Teloken1 | N | 63.6 | 8.89 | 100 | N |
| Teloken2 | N | 60.4 | 6.63 | 75.5 | N |

## TableS2. The use of neoadjuvant or adjuvant treatment in the cohorts.

| Study | Neoadjuvant therapy or adjuvant therapy before BCR |
| --- | --- |
| Asuncion | 19% with ADT |
| Billis | 0 until BCR |
| Chun | 0 (excluded) |
| Falzarano | 0 (excluded) |
| Iremashvili | 0 (excluded) |
| Kim1 | Not reported |
| Kim2 | Not reported |
| Lee | 0 (excluded) |
| Magheli | 0 (excluded) |
| Mygatt | Not reported |
| Sakai | 0 (excluded) |
| Sato | The day of initial adjuvant treatment was defined as the day of BCR. |
| Shin | 0 (excluded) |
| Takamatsu | 0 (excluded) |
| Teloken1 | 0 (excluded) |
| Teloken2 | 0 (excluded) |

## TableS3. Methodological quality of included studies.

| Cohort studies | Selection | | | | Comparability | Outcome | | | Total score |
| --- | --- | --- | --- | --- | --- | --- | --- | --- | --- |
|  | Representativeness of exposed cohort | Selection of non-exposed cohort | Ascertainment of exposure | Presentation of outcome at start | Control for important factor^a^ | Assessment of outcome | Time of follow-up^b^ | Adequacy of follow-up |  |
| Asuncion^[21]^ | 1 | 1 | 1 | 1 | 0 | 1 | 1 | 1 | 7 |
| Billis^[22]^ | 1 | 1 | 1 | 1 | 1 | 1 | 1 | 1 | 8 |
| Chun^[23]^ | 1 | 1 | 1 | 1 | 0 | 1 | 1 | 1 | 7 |
| Iremashvili^[25]^ | 1 | 1 | 1 | 1 | 2 | 1 | 1 | 1 | 9 |
| Kim^[26]^ | 0 | 0 | 1 | 1 | 1 | 1 | 0 | 0 | 4 |
| Lee^[27]^ | 1 | 1 | 1 | 1 | 2 | 1 | 1 | 1 | 9 |
| Magheli^[28]^ | 1 | 1 | 1 | 1 | 1 | 1 | 1 | 1 | 8 |
| Mygatt^[29]^ | 1 | 1 | 1 | 1 | 1 | 1 | 1 | 1 | 8 |
| Sakai^[30]^ | 1 | 1 | 1 | 1 | 2 | 1 | 1 | 1 | 9 |
| Sato^[31]^ | 1 | 1 | 1 | 1 | 2 | 1 | 1 | 1 | 9 |
| Shin^[32]^ | 1 | 1 | 1 | 1 | 1 | 1 | 0 | 1 | 7 |
| Takamatsu^[33]^ | 1 | 1 | 1 | 1 | 2 | 1 | 1 | 1 | 9 |
| Teloken1^[34]^ | 1 | 1 | 1 | 1 | 1 | 1 | 1 | 1 | 8 |
| Teloken2^[34]^ | 1 | 1 | 1 | 1 | 0 | 1 | 1 | 1 | 7 |

| Case-control studies | Selection | | | | Comparability | Exposure | | | Total score |
| --- | --- | --- | --- | --- | --- | --- | --- | --- | --- |
|  | Adequate definition of cases | Representativeness of cases | Selection of controls | Definition of control | Control for important factor | Ascertainment of exposure | Same method | Non-response rate |  |
| Falzarano^[24]^ | 1 | 1 | 1 | 1 | 2 | 1 | 1 | 1 | 9 |

^a^ Studies in which the T stage and Gleason grade were both balanced or adjusted was judged as 2 scores. If one of the T stage and the Gleason grade was balanced or adjusted, the study was judged as 1 score. If none of them was balanced or adjusted, the study was a 0 score.

^b^ Studies with a median follow-up of fewer than 30 months were judged as 0 scores, and otherwise, 1 score.

## TableS4. Sensitivity analysis of pooled RRs with 95% CI with certain study omitted

| Study omitted | Pooled RRs | 95% CI | |
| --- | --- | --- | --- |
| Asuncion | 0.80 | 0.69 | 0.93 |
| Billis | 0.80 | 0.68 | 0.93 |
| Chun | 0.79 | 0.68 | 0.92 |
| Falzarano | 0.79 | 0.68 | 0.92 |
| Iremashvili | 0.81 | 0.70 | 0.94 |
| Kim1 | 0.75 | 0.61 | 0.93 |
| Kim2 | 0.77 | 0.66 | 0.90 |
| Lee | 0.81 | 0.70 | 0.94 |
| Magheli | 0.80 | 0.69 | 0.94 |
| Mygatt | 0.78 | 0.67 | 0.91 |
| Sakai | 0.78 | 0.67 | 0.91 |
| Sato | 0.83 | 0.72 | 0.95 |
| Shin | 0.81 | 0.70 | 0.93 |
| Takamatsu | 0.81 | 0.70 | 0.94 |
| Teloken1 | 0.81 | 0.69 | 0.94 |
| Teloken2 | 0.75 | 0.65 | 0.87 |
| Combined | 0.79 | 0.69 | 0.92 |

## FigureS1. Forest plot of the association between transition zonal origin and biochemical recurrence. Data are stratified by the Gleason grade group: 1, Grg≥2 ratio < 80%; 2, Grg≥2 ratio ≥ 80%; 3, Grg≥2 ratio unavailable.

##

## FigureS2. Sensitivity analysis plot showing estimated pooled RRs with 95% CI with certain study omitted.

##

## FigureS3. Funnel plot with pseudo 95% confidence limits.
